# Improved Weighted Non-Local Mean Filtering Algorithm for Laser Image Speckle Suppression

**DOI:** 10.3390/mi14010098

**Published:** 2022-12-30

**Authors:** Jin Cheng, Yibo Xie, Shun Zhou, Anjiang Lu, Xishun Peng, Weiguo Liu

**Affiliations:** 1 School of Optoelectronic Engineering, Xi’an Technological University, Xi’an 710000, China; 2 College of Big Data and Information Engineering, Guizhou University, Guiyang 550000, China

**Keywords:** structured light, speckle noise, non-local mean filter, denoising, image processing

## Abstract

Laser speckle noise caused by coherence between lasers greatly influences the produced image. In order to suppress the effect of laser speckles on images, in this paper we set up a combination of a laser-structured light module and an infrared camera to acquire laser images, and propose an improved weighted non-local mean (IW-NLM) filtering method that adopts an SSI-based adaptive h-solving method to select the optimal h in the weight function. The analysis shows that the algorithm not only denoises the laser image but also smooths pixel jumps in the image, while preserving the image details. The experimental results show that compared with the original laser image, the equivalent number of looks (ENL) index of the IW-NLM filtered image improved by 0.80%. The speckle suppression index (SSI) of local images dropped from 4.69 to 2.55%. Compared with non-local mean filtering algorithms, the algorithm proposed in this paper is an improvement and provides more accurate data support for subsequent image processing analysis.

## 1. Introduction

Lasers are widely used in imaging systems, scanning systems, lighting, etc. [1], because of their high monochromatism, wide color range, high brightness, and long life. The laser imaging system built in this paper is essentially composed of a transmitting unit and a receiving unit [2]. Its working principle is that the laser beam at the transmitting port scans the target object, and the receiving port receives the reflected laser radiation to generate a group of continuous signals and restore them to obtain the image of the target scene. When the laser is emitted to the object’s surface, laser speckles are formed due to the interaction of coherent light irradiation, which seriously affects the effectiveness of laser imaging [3]. The speckles are characterized by highly random image patterns with different imaging distances. Therefore, it is very important to eliminate the speckle noise in laser images. In order to eliminate or reduce speckle, the current method is mainly to use hardware optical systems and software image processing. Hardware improvements are usually made to the internal parts of the optical system. Common materials include ground glass [4], vibrating fiber [5], and rotating micro-lens [6], which are usually complex and highly demanding for the system. So, many scholars have studied how to eliminate or suppress the speckle noise of images by using image noise reduction algorithms in software.

Laser speckles seriously impact active illumination systems, so there is an urgent need to study filtering algorithms for software image processing to suppress the noise in laser images. Image denoising algorithms are mainly divided into spatial domain, transform domain, and neural network denoising algorithms [7]. The image-denoising algorithm based on the transform domain is not widely used in laser image denoising due to its complex computation and difficulty preserving edge details. Although neural networks have become a research hotspot in recent years, the datasets in neural networks are difficult to obtain, and the data training speed can be slow. The common image filtering algorithms based on spatial domain include mean filter [8], median filter [9], Gaussian filter [10], and bilateral filter [11]. However, these algorithms can only remove specific noise effectively, so they are not suitable for speckle removal in laser images. Filtering algorithms based on the frequency domain include ideal high/low-pass filter [12], Butterworth high/low-pass filter [13], and Gaussian high/low-pass filter [14]. With continuous research by scholars at home and abroad, filtering algorithms are emerging one after the other, such as guided filter [15] calculated iteratively, homomorphic filter [16] combining frequency filter and spatial grey transformation, and various fusion filtering algorithms.

It is not satisfactory that the denoising effects of these filtering algorithms for laser images and the detailed information of images may be lost, as this limits the application of laser imaging systems. So, an improved weighted filtering algorithm based on a non-local mean filter (IW-NLM) is proposed in this paper to improve the quality of laser images. By exploiting the redundant information of the image, the NLM [17] can remove the noise and retain as many high-frequency details of the image as possible. This improved algorithm can effectively suppress speckle noise and preserve more details. An adaptive h-solving method is used to select suitable parameters for different laser images to enhance the quality of laser images. The weight function is modified to adjust the magnitude of the weights at different Euclidean distances.

## 2. Laser Image Acquisition System

**Figure 1:**
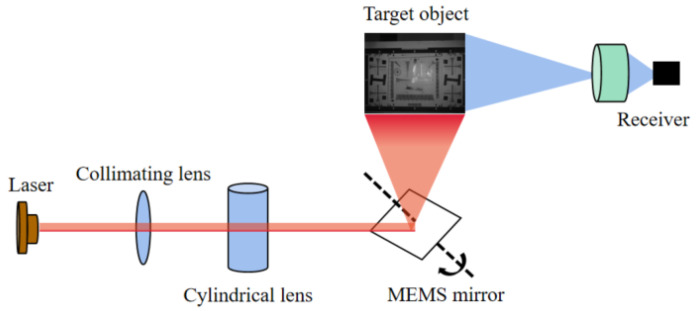
Schematic diagram of laser-structured light projector and imaging receiver.

The laser image used in this paper was obtained by a device containing a laser-structured light projector and receiver, according to the principle shown in Figure 1. The light emitted from the laser is collimated through a collimating lens, passes through a cylindrical lens, and is illuminated on the MEMS mirror. By controlling the switching of the laser and the scanning angle of the MEMS mirror, the space projection of high-speed and high-resolution coded structured light can be realized. The receiver acquires a laser image containing speckle noise [18] (generated by interference from a part of the laser reflected from the target).

**Figure 2:**
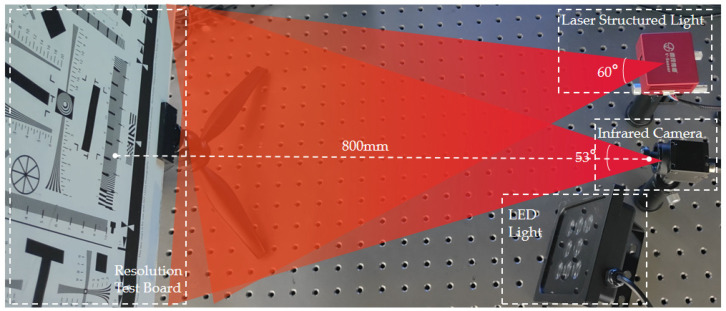
Laser image acquisition system can acquire laser image with speckle noise.

According to the principle in Figure 1, the laser image acquisition system was built as shown in Figure 2. It is composed of an industrial camera and a laser-structured light module (the device in Figure 1 is often used in 3D image reconstruction applications [19], but due to the laser coherence, the image captured by the camera has speckle noise, which affects the reconstruction accuracy). The laser-structured light module (MSC-PO8508, Wuxi V-Sensor Technology Co., Ltd., Wuxi, China) uses a light source with a wavelength of 850 ± 5 nm, and the camera uses a 1.3-megapixel near-infrared camera; its parameters are shown in Table 1. Spatial resolution is often used to evaluate the imaging capability of laser imaging systems, and it is calculated based on the mapping relationship of pixel size in space using Equation (1). When shooting distance *l* is 800 mm, the spatial resolution *d* is 640 μm (when the shooting distance is ≥800 mm, the view of the infrared camera can cover the measured target). Therefore, when evaluating the clarity of the measured object, we focus on the information with feature size ≥640 μm.

(1) $$d=\frac{l\times D_{pix}}{f}$$

In the equation, *l* is the shooting distance, *D_pix_* is the camera’s pixel size, and *f* is the camera’s focal length.

**Figure 3:**
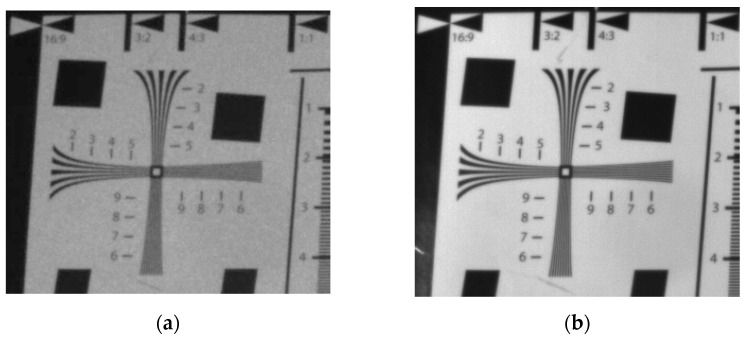
Comparison of several local magnification images (determination of visual resolution): (**a**) local magnification of original laser image with speckle noise; (**b**) local magnification of LED image without speckle noise.

Figure 2 shows the shooting environment of the laser image acquisition system, which includes a laser-structured light module, infrared camera, LED light, and resolution test board. There is a filter lens in front of the infrared camera, which is used to absorb the infrared part of solar radiation and at the same time filter the light reflected on the lens to improve the image’s sharpness. The target of the shot is the ISO12233 resolution test board, which is usually used to check the camera resolution. First, we turned on the laser structured light module, turned off the LED light, and shot the image of the local area, as shown in Figure 3a; then, we turned off the light module, turned on the LED light, and shot the image of the local area, as shown in Figure 3b. A local view of the resolution test board is shown in Figure 3a, which does not clearly distinguish between black and white line pairs due to the presence of noise, so image filtering is required to eliminate speckles and improve spatial resolution.

According to the acquisition system, the laser image is obtained, and speckle noise in the image affects its quality. However, there is no laser noise in the LED image. In order to facilitate observation, the visual resolution measurement part of the four-cornered cross in the image was enlarged (Figure 3), which is part of the wedge line test of the resolution test board. The laser image in Figure 3a has speckle noise in the background, which leads to blurred frequency lines; in comparison, the LED image in Figure 3b has clear details. In this paper, an improved weighted non-local mean (IW-NLM) filtering algorithm is proposed in Section 3 to suppress the speckle noise of laser images.

## 3. Improved Weighted Non-Local Mean (IW-NLM) Filtering Algorithm

An image is essentially a two-dimensional signal, and image filtering is performed to attenuate or eliminate the adverse impact of noise and improve the image’s quality. This paper proposes an IW-NLM filtering algorithm for noise removal from laser images and compares it with the non-local mean (NLM) filtering algorithm.

### 3.1. Non-Local Mean (NLM) Filtering Algorithm

NLM filtering weights the pixel values according to the similarities between pixel regions. Using the global information of the image can better preserve the detailed information. The NLM algorithm iterates over all pixels of the image and searches for similar regions to the region centered on each pixel. The obtained similarity is proportional to weight; the per-pixel value is the sum of the products of all pixels in the search window and their corresponding weights [20].

**Figure 4:**
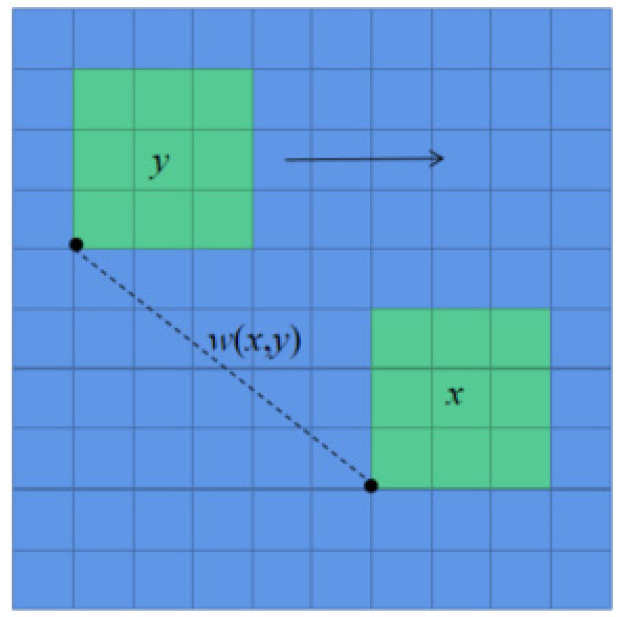
Schematic diagram of non-local mean filter.

Two windows are defined: the search window (size: d × d), and the neighborhood window (size: D × D). The neighborhood window is traversed in the search window, and the pixel weight value is obtained by solving the similarity degree between neighbors. In Figure 4, the blue box centered on pixel *x* is regarded as the search window, and the green window centered on pixel y is traversed within the large window. The similarity between the small green windows centered on *x* and *y* is calculated, and this value is taken as the weight of *y*.

In the image, each pixel point *v*(*x*) is ultimately represented by the sum of the grey value for each pixel point *v*(*y*) in the search box multiplied by its corresponding weight value *w*(*x*, *y*). This relationship is represented by Equation (2):

(2) $$v(x)=\sum_{y}w(x,y)v(y)$$

where *w* (*x*, *y*) is the similarity between pixels *x* and *y*, whose value is represented by the Gaussian weighted Euclidean distance between neighborhood *N*(*x*) and *N*(*y*) centered on *x* and *y*:

(3) $$w\left(x,y\right)=\frac{1}{Z\left(x\right)}exp(-\frac{|\left|N\left(x\right)-N\left(y\right)\right|{|}^{2}}{h^{2}})$$

The Euclidean distance between two neighborhoods and the normalized parameters are respectively expressed as:

(4) $$||N(x)-N(y)|{|}^{2}=\frac{1}{d^{2}}\sum_{||z|{|}_{\infty }\leq ds}||v(x+z)-v(y+z)|{|}^{2}$$

(5) $$Z\left(x\right)=\underset{y}{\sum }exp(-\frac{|\left|N\left(x\right)-N\left(y\right)\right|{|}^{2}}{h^{2}})$$

*Z*(*x*) is the normalized coefficient and h is the smoothing parameter, which controls the strength of noise reduction effect. The larger h is, the gentler the change of the Gaussian function and the greater the noise reduction intensity, and the image can become blurred. With smaller h, the noise reduction intensity is smaller and more edge details are preserved, but at the same time, more noise is retained. Therefore, the magnitude of h varies with the noise in different images.

The conventional NLM algorithm does not consider the image’s noise type; only the multiplicative noise of image is processed, the additive noise processing is not analyzed. Therefore, in the next section we propose an improved non-local mean filtering method for laser images.

### 3.2. Improved Non-Local Mean (I-NLM) Filtering Algorithm

**Figure 5:**
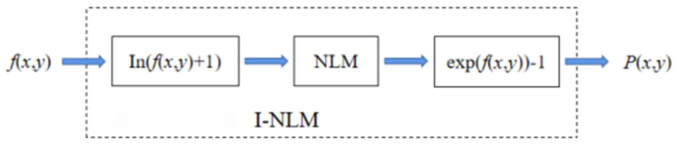
Flowchart of I-NLM filtering process.

The laser image acquisition system is analyzed in Section 2 of this paper. Laser speckle noise is a type of multiplicative noise [21], expressed as bright or dark patches in the image. The general process of I-NLM filtering, based on the excellent denoising performance of NLM for additive noise, is shown in Figure 5. First, the multiplicative noise is converted into additive noise by logarithmic processing of the original laser image. Then the NLM filtering algorithm is used to suppress speckle noise in the high-frequency information and preserve the image’s details. Finally, the processed image is exponentially transformed to obtain a filtered image.

I-NLM is used to eliminate speckle noise according to Figure 5. However, by analyzing the principle of NLM, we find that the size of h affects the filtering effect of laser images and the degree of detail preservation. Therefore, an adaptive approach is used to select h.

### 3.3. SSI-Based Adaptive h-Solving Method

The h of NLM has a certain influence on the filtering results of images, thus the main content of this section is how to choose the appropriate h for a better filter effect according to the speckle suppression index (SSI) values of different local images. Due to the differences between laser images, an adaptive approach is used to solve for the appropriate h corresponding to the current laser image. Meanwhile, to ensure that image noise can be better eliminated, SSI is used to evaluate the suppression effect of the local image. Based on this, this paper proposes an SSI-based adaptive h-solving method. We get the h value when the SSI is taken to be the smallest; see Equation (8) in Section 4.

**Figure 6:**
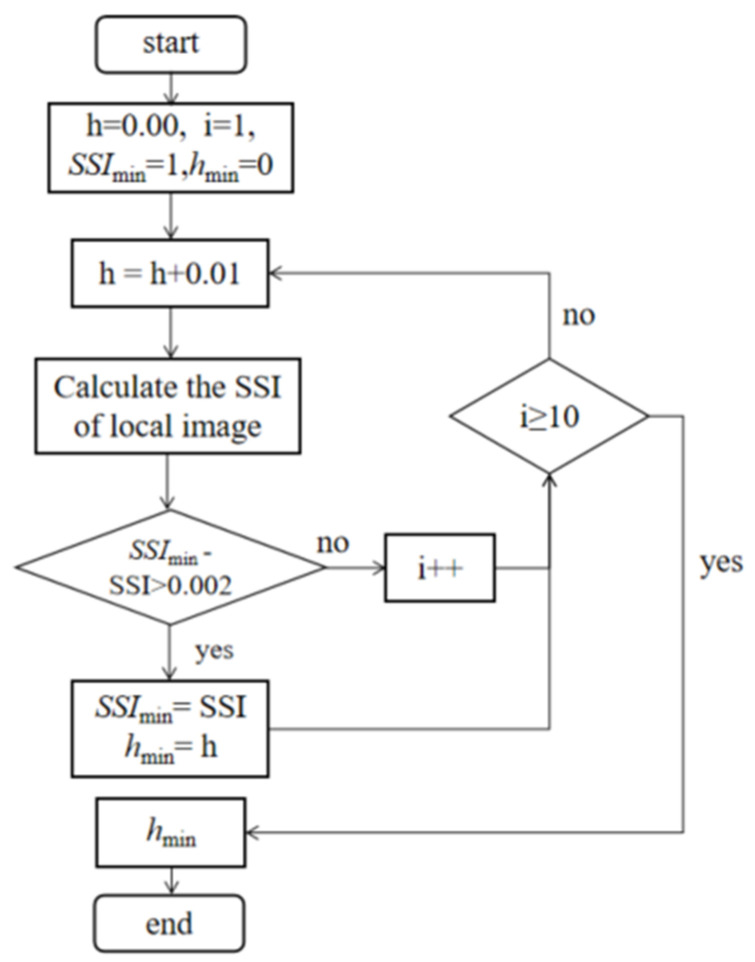
SSI-based adaptive h-solving method.

The NLM parameter h increases from 0.01, with steps of 0.01. To prevent h from being too large, which would result in detailed information not being retained, *h*_min_ is updated when the difference between *SSI*_min_ and SSI is greater than 0.002. There is a timer in this method that requires *SSI*_min_ to be compared nine times, and if the *h*_min_ value is not updated, *h*_min_ is returned after the ninth time. The value is chosen to remove speckle noise and preserve the image’s detailed information as much as possible. The specific process is shown in Figure 6.

**Figure 7:**
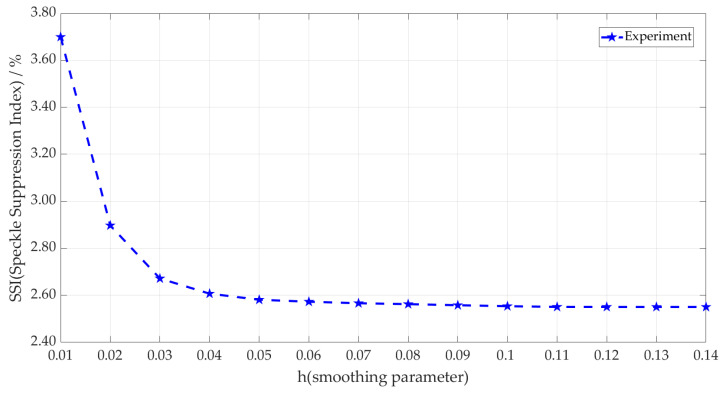
Optimal h-values of I-NLM using adaptive h-solving method.

Figure 7 shows the processing of the laser image in Figure 10a using the I-NLM method to find a suitable h value according to the adaptive method. It can be seen that the SSI value of local images decreases rapidly when h < 0.05 and changes very little when h > 0.05. The h parameter represents the discreteness degree of the data. If h is small, the central coefficients of the generated template are larger and the surrounding coefficients are smaller, and the smoothing effect is not very obvious; on the contrary, if h is large, the individual coefficients of the generated template do not differ much and the smoothing effect on the image is more obvious. Therefore, the optimal h value of I-NLM is 0.05 by the SSI-based adaptive h-solving method, which ensures that speckle noise is effectively suppressed and the detailed information in the image is preserved to the maximum extent.

### 3.4. Optimizing the Weighting Function of IW-NLM

Analyzing the weight function in I-NLM shows that when the difference between two pixels is too large, it is still in the assignment of the weight value; in fact, when the two are too large, the weight value should converge to 0 to prevent the introduction of too much noise error. When the pixel values are close, the function is too sensitive to distance, leading to a too-large gradient in the weight. Both of these occurrences will affect the effect of noise reduction. Therefore, the weighting function is optimized to have high weights when the distance is small and low weights when the distance is large to reduce the introduction of noise.

In response to the above, we increase the exponential coefficient of the Euclidean distance ||*N*(*x*)-*N*(*y*)|| so that the gradient of *w*’(*x*, *y*) becomes smaller when the Euclidean distance is similar, and *w*’(*x*, *y*) quickly converges to 0 when the Euclidean distance is too far, as shown in Equation (6):

(6) $$w^{\prime }\left(x,y\right)=\frac{1}{Z\left(x\right)}exp(-\frac{{\left(|\left|N\left(x\right)-N\left(y\right)\right|{|}^{2}\right)}^{2}}{h^{2}})$$

**Figure 8:**
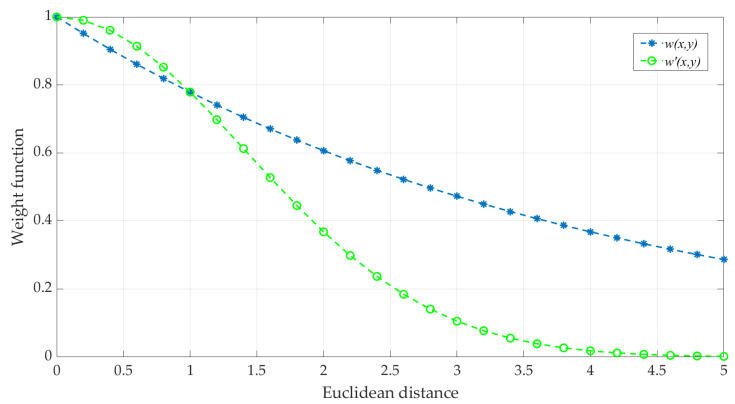
Comparison graph of weight function before and after optimization.

The graph comparing before and after the weight function is optimized is shown in Figure 8. Here we let h be a constant value of 2.0, and get the graph before and after modifying the weight function. The graph also proves that the weight value decreases more slowly when the distance is closer to 0.0–1.0; when the distance is 1.0–3.5, the weights fall faster; and when the distance is greater than about 4.0, the weights converge to 0.

**Figure 9:**
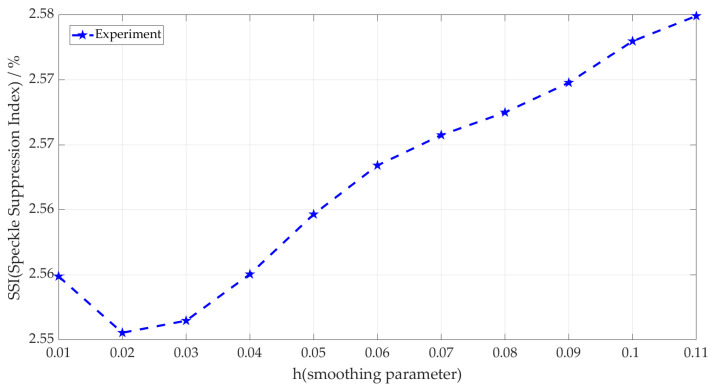
Optimal h value of optimized weight function IW-NLM using adaptive h-solving method.

**Figure 10:**
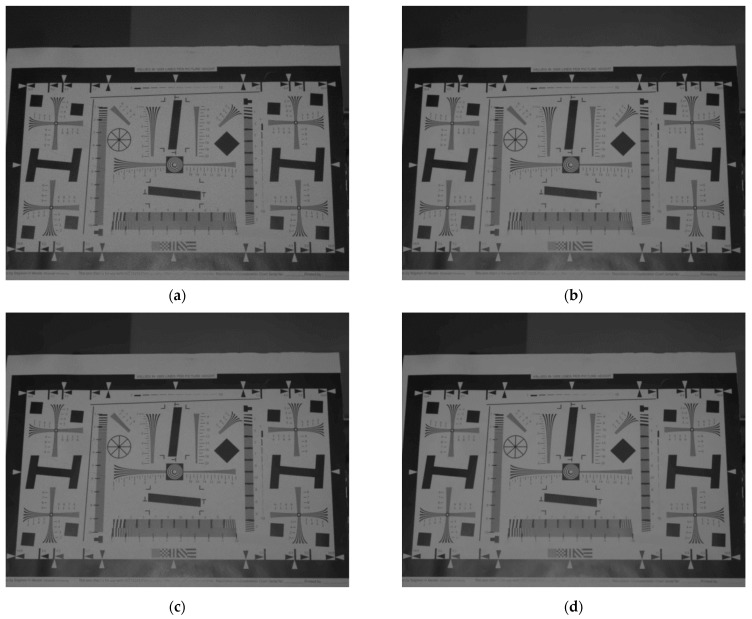
Comparison results of several images: (**a**) original laser image with speckle noise; (**b**) non-local mean filter image; (**c**) improved non-local mean filter image; (**d**) improved weighted non-local mean filter image.

Similarly, Figure 9 shows the processing of the laser image in Figure 10a using the IW-NLM method to find a suitable h value according to the adaptive approach. In the figure, the minimum SSI value of the local image of optimized weight function IW-NLM was obtained at 0.02; the optimal h value after optimization was 0.02, obtained by the adaptive h-solving method of local image SSI.

The IW-NLM filter introduced here is used to suppress the high-frequency noise information in images captured by a laser imaging system and improve the image quality.

## 4. Results and Evaluation

To verify the effectiveness of the IW-NLM filtering algorithm proposed in this paper, the equivalent number of looks (ENL) [22] and speckle suppression index (SSI) values [23] are used to compare the image results of filtering algorithms and evaluate the algorithms’ ability to suppress speckle noise.

The equivalent number of looks in the evaluation index reflects the speckle noise removal ability. The larger the ENL value, the stronger the ability to remove spot noise. It is expressed by Equation (7):

(7) $$ENL={\left(\frac{\mu }{\sigma }\right)}^{2}$$

where *μ* and *σ*, respectively, represent the mean and standard deviation of the image, and the size of ENL is proportional to the level of image quality.

The speckle suppression index is used to evaluate the speckle suppression ability of the filtering algorithm. The smaller the value, the stronger the inhibition ability and the better the image quality.

(8) $$SSI=\frac{1}{MN}\sum_{i=1}^{M}\sum_{j=1}^{N}\frac{\sigma (i,j)}{\mu (i,j)}$$

In the equation, *σ* (*i*, *j*) and *μ* (*i*, *j*), respectively, represent the standard deviation and average element grey value in the measurement window.

The resolution test board image was processed using the conventional NLM, I-NLM, and IW-NLM filtering algorithms, as shown in Figure 10. The evaluation metrics were also used to compare the denoising performance of the algorithms and the degree of image detail retention.

**Figure 11:**
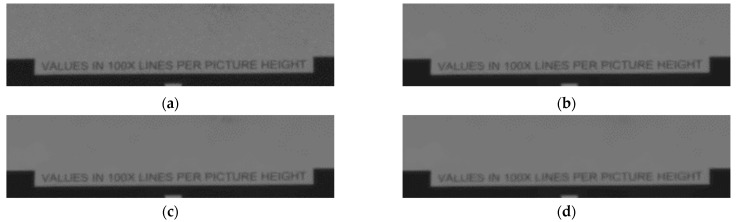
Comparison results of several local magnification images (determination of visual resolution): (**a**) local magnification of original laser image with speckle noise; (**b**) local magnification of non-local mean filter image; (**c**) local magnification of improved non-local mean filter image; (**d**) local magnification of improved weighted non-local mean filter image.

To verify the ability of the algorithm to retain image details, the local background part of the resolution test board image was enlarged, as shown in Figure 11. The resolution of the enlarged portion is 360 × 90, which is directly observed.

The laser image in Figure 11a has large speckle noise in the local background. Figure 11b shows the image after NLM filtering; although some speckle noise is eliminated, there is still local noise present, affecting the image quality. Figure 11c shows the result of using NLM by converting multiplicative noise to additive noise and suppressing the noise in the background. Figure 11d shows the filtering after optimizing the weighting function. The result not only retains the detailed information as much as possible, but also better eliminates the black and white speckle noise in the background. This is because the NLM algorithm relates pixels to each other, eliminating jumpy pixel points and smoothing out the overall pixel values.

**Figure 12:**
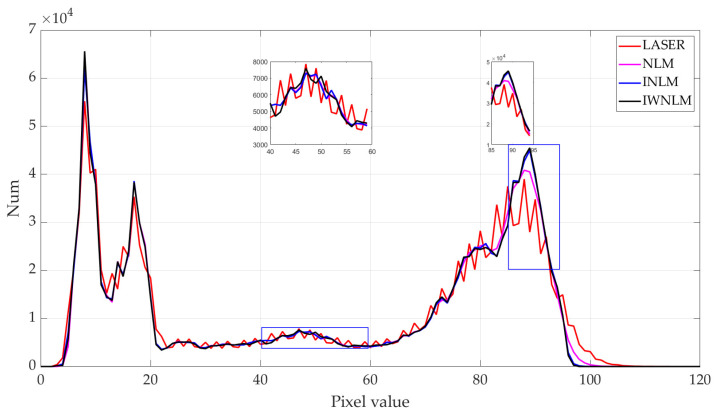
Histogram distribution of laser image and filtered images.

A histogram distribution generally describes the overall distribution of image pixels, as a table representing the number of pixels with a certain value in an image [16]. In the histogram, there are 256 abscissa values to represent the pixel values (the pixel distribution is mainly concentrated below 120 pixels, so values above 120 are not shown in the graph for a better display), and the vertical axis represents the distribution. The histogram distribution of the original laser image and the filtered image is shown in Figure 12. The macroscopic display shows that the pixel distribution in four images is mainly concentrated between 0 and 100, and in the laser image the pixel distribution is unstable and fluctuates up and down. By local magnification, it can be observed that the pixel distribution of the three filter images is more stable. The black curve in the graph indicates that the proposed IW-NLM method makes a natural transition between pixels without a large prominent distribution.

**Figure 13:**
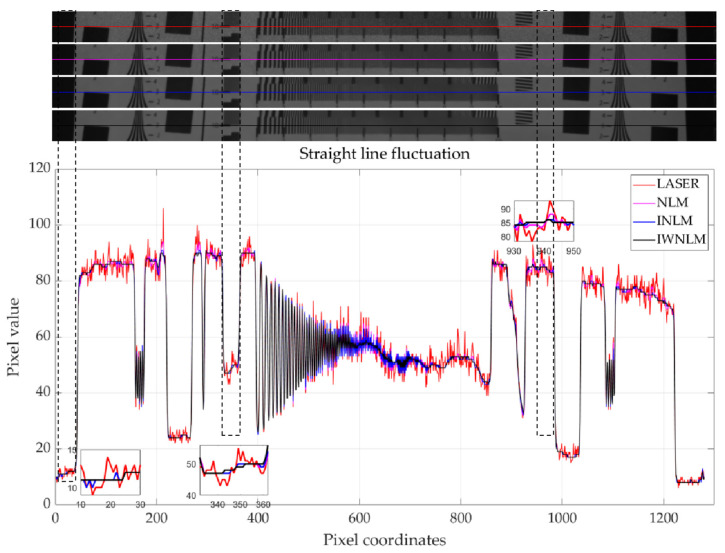
Linear fluctuations of laser image and filtered image.

To better describe the suppression of speckle noise in laser images by the method proposed in this paper, the fluctuations were observed by taking a straight line to the image. In Figure 13, the original laser image, non-local mean filtered image, improved non-local mean filtered image, and improved weighted non-local mean filtered image are described linearly with red, pink, blue, and black lines corresponding to each image, respectively. First is the black border of the test board, where the red line of the original laser image fluctuates strongly, the blue line fluctuates slightly, and the black and pink lines do not fluctuate. Next is the frequency line of the MTF test in the resolution test board, which is the same, with similar border fluctuation, and it can be observed in the figure that the blue line in the border conversion has a more obvious jump, while the black line conversion is gentler. Third is the white background of the test board, which can be observed with the first two differences, showing that the pink line fluctuation increased, while the black and blue lines are relatively smooth. When we observe the resolution board’s horizontal frequency line, the fluctuation of the black line gradually diminishes, but there is still strong regularity, with no large jump, while the red line shows irregularly large jumps and the blue line fluctuates with little regularity. By observing Figure 13 and analyzing the fluctuations of several images in a certain line, it can be seen that the regularity and smoothness of the IW-NLM image are optimal.

ENL is an evaluation metric that measures the smoothness of a homogeneous area of an image. SSI is used to evaluate the suppression of speckle noise in laser images, and the smaller the value, the better the suppression effect. These two metrics are used to evaluate the quality of images. In Table 2, the ENL values of the four global images increase from top to bottom; the best one reaches 0.7199. The speckle noise value of the local background of the four images decreases in order, and the scattering coefficient of the IW-NLM local image decreases to 2.55%. The method in this paper represents a small but valuable improvement over NLM. Because of this improvement, the edge details can be better preserved, especially in terms of precision 3D measurement, and this small improvement will bring high value. Compared with the traditional NLM algorithm, the time efficiency difference with the proposed algorithm is not significant; the time efficiency issue will be considered in subsequent research to further optimize the algorithm.

According to the results of image, histogram, and linear fluctuation analysis and the data visualization mentioned above, the IW-NLM algorithm proposed in this paper eliminates speckle noise better and retains the detailed information of the image. The adaptive h value method makes the filtering effect of the image optimal, and the adjustment of the weight function makes the distribution of the weights more reasonable. The final image results prove that the IW-NLM image quality is improved and the data result is optimal.

## 5. Conclusions

In order to solve the problem of speckle noise in images acquired by laser imaging systems, in this paper we built a laser image acquisition system combining a laser-structured light module and an infrared camera, and proposed an improved weighted non-local mean filtering algorithm to suppress the speckle noise in the images. ENL and SSI indexes were used to evaluate the speckle suppression effect of all methods. The experimental results show that the IW-NLM algorithm can eliminate the speckle noise of laser images, smooth the huge jumps of image pixels while retaining the detail information, and obtain higher-quality laser images than the NLM algorithm. Compared with the original laser image, the ENL of the IW-NLM image is improved by 0.80%, and the SSI of the local laser image is reduced from 4.69 to 2.55%. The research in this paper provides more accurate data support for subsequent image processing, which can be applied to laser imaging, laser scanning display, and other fields.

## Figures and Tables

**Table 1:** Parameters of laser-structured light module and infrared camera.

Item	Laser-Structured Light Module	Infrared Camera
Band (nm)	850 ± 5	800–900
Resolution	1280 × 1	1280 × 1024
Working distance (mm)	500–1000	Manual focus
FOV (H × V)	60° × 48°	53° × 40°
Pixel size (μm)	/	4.8
Focal length (mm)	/	6
F number	/	2.8

**Table 2:** Comparison of image quality evaluation indexes.

Image	ENL	SSI	Time (s)
Original laser image	0.7142	4.69%	/
NLM filtered image	0.7185	2.74%	8.488
I-NLM filtered image	0.7188	2.58%	9.520
IW-NLM filtered image	0.7199	2.55%	11.643

## Data Availability

Not applicable.

## References

[1] Wu J., Yin K., Xiao S., Wu Z., Zhu Z., Duan J., He J. (2021). Laser Fabrication of Bioinspired Gradient Surfaces for Wettability Applications. Adv. Mater. Interfaces.

[2] Wang Q., Li Q., Chen Z., Sun J., Yao R. (2008). Range image noise suppression in laser imaging system. Opt. Laser Technol..

[3] Du E., Shen S., Qiu A., Chen N. (2022). Confocal laser speckle autocorrelation imaging of dynamic flow in microvasculature. Opto Electron. Adv..

[4] Kim H., Shim Y., Lee K., Han J., Yi C., Kim Y. (2007). Persistent pulmonary nodular ground-glass opacity at thin-section CT: Histopathologic comparisons. Radiology.

[5] Fedorchenko A., Stachiv I., Wang A. (2009). The optical viscometer based on the vibrating fiber partially submerged in fluid. Sens. Actuators B Chem..

[6] Mogi T., Hatakeyama K., Taguchi T., Wake H., Tanaami T., Hosokawa M., Tanaka T., Matsunaga T. (2011). Real-time detection of DNA hybridization on microarray using a CCD-based imaging system equipped with a rotated microlens array disk. Biosens. Bioelectron..

[7] Wei Y. (2020). Three-dimensional laser image-filtering algorithm based on multi-source information fusion and adaptive offline fog computing. Multimed. Syst..

[8] Thanh D., Engínoğlu S. (2019). An iterative mean filter for image denoising. IEEE Access.

[9] Boateng K., Asubam B., Laar D. (2012). Improving the effectiveness of the median filter. Int. J. Electron. Commun. Eng..

[10] Pan B. (2013). Bias error reduction of digital image correlation using Gaussian pre-filtering. Opt. Lasers Eng..

[11] Lu H., Li Y., Serikawa S. Underwater image enhancement using guided trigonometric bilateral filter and fast automatic color correction. Proceedings of the 2013 20th IEEE International Conference on Image Processing.

[12] Liu T., Zhang W., Yan S. (2015). A novel image enhancement algorithm based on stationary wavelet transform for infrared thermography to the de-bonding defect in solid rocket motors. Mech. Syst. Signal Process..

[13] Sciacchitano A., Scarano F. (2014). Elimination of PIV light reflections via a temporal high pass filter. Meas. Sci. Technol..

[14] Liang J., Wu S., Kohn R., Becker M., Heinzen D. (2012). Grayscale laser image formation using a programmable binary mask. Opt. Eng..

[15] Yan X., Qin H., Li J., Zhou H., Yang T. (2016). Multi-focus image fusion using a guided-filter-based difference image. Appl. Opt..

[16] Fan Y., Zhang L., Guo H., Hao H., Qian K. (2020). Image Processing for Laser Imaging Using Adaptive Homomorphic Filtering and Total Variation. Photonics.

[17] Li W., Guo S., Zhai Y., Liu F., Lai Z., Han S. (2021). Denoising of the multi-slit streak tube imaging LiDAR system using a faster non-local mean method. Appl. Opt..

[18] Redding B., Choma M., Cao H. (2012). Speckle-free laser imaging using random laser illumination. Nat. Photonics.

[19] Zhu Z., Yang J., Wang X., Qi G., Wu C., Fan H., Qi L., Dong J. Rotation Axis Calibration of Laser Line Rotating-Scan System for 3D Reconstruction. Proceedings of the 2020 11th International Conference on Awareness Science and Technology.

[20] Wang J., Guo Y., Ying Y., Liu Y., Peng Q. Fast non-local algorithm for image denoising. Proceedings of the 2006 International Conference on Image Processing.

[21] Khan M., Altalbe A. (2022). Experimental evaluation of filters used for removing speckle noise and enhancing ultrasound image quality. Biomed. Signal Process. Control.

[22] Cui Y., Zhou G., Yang J., Yamaguchi Y. (2011). Unsupervised estimation of the equivalent number of looks in SAR images. IEEE Geosci. Remote Sens. Lett..

[23] Kulkarni S., Kedar M., Rege P. Comparison of Different Speckle Noise Reduction Filters for RISAT-1 SAR Imagery. Proceedings of the 2018 International Conference on Communication and Signal Processing.

